# Modern World Applications for Nano-Bio Materials: Tissue Engineering and COVID-19

**DOI:** 10.3389/fbioe.2021.597958

**Published:** 2021-05-14

**Authors:** Elda M. Melchor-Martínez, Nora E. Torres Castillo, Rodrigo Macias-Garbett, Sofia Liliana Lucero-Saucedo, Roberto Parra-Saldívar, Juan Eduardo Sosa-Hernández

**Affiliations:** Tecnologico de Monterrey, School of Engineering and Sciences, Monterrey, Mexico

**Keywords:** tissue engineering, COVID-19 therapy, biomaterials, multifunctional entities, drug delivery system, fabrication strategies, biomedical applications

## Abstract

Over the past years, biomaterials-based nano cues with multi-functional characteristics have been engineered with high interest. The ease in fine tunability with maintained compliance makes an array of nano-bio materials supreme candidates for the biomedical sector of the modern world. Moreover, the multi-functional dimensions of nano-bio elements also help to maintain or even improve the patients’ life quality most securely by lowering or diminishing the adverse effects of in practice therapeutic modalities. Therefore, engineering highly efficient, reliable, compatible, and recyclable biomaterials-based novel corrective cues with multipurpose applications is essential and a core demand to tackle many human health-related challenges, e.g., the current COVID-19 pandemic. Moreover, robust engineering design and properly exploited nano-bio materials deliver wide-ranging openings for experimentation in the field of interdisciplinary and multidisciplinary scientific research. In this context, herein, it is reviewed the applications and potential on tissue engineering and therapeutics of COVID-19 of several biomaterials. Following a brief introduction is a discussion of the drug delivery routes and mechanisms of biomaterials-based nano cues with suitable examples. The second half of the review focuses on the mainstream applications changing the dynamics of 21st century materials. In the end, current challenges and recommendations are given for a healthy and foreseeable future.

## Introduction

Nano-biomaterials have become a useful tool for medical applications for several reasons that include compatibility and novel effects due to nanoscale. The possibilities to add biomaterials to the development of nanostructures have opened the door for innovative applications in several fields (Karagkiozaki et al., 2012; Elmowafy et al., 2019). One of the most important is for modern medicine with exceptionally complex problems to solve. Modern medicine has incorporated nanotechnology into two major topics of general concern, tissue engineering and novel viruses, which were chosen in this review due to significant cases and impact (Torres-Sangiao et al., 2016; van Rijn and Schirhagl, 2016; Kapat et al., 2020).

Biomaterials compromise the group of substances that either are produced by living organisms or highly compatible. In this matter, numerous research work has been done to test and use the materials in modern medicine. An extensive list of medical applications can be found elsewhere (Nune and Misra, 2016; Tang et al., 2016; Gim et al., 2019; Luzi et al., 2019; Arango-Ospina et al., 2020; Song et al., 2020). The property of biocompatibility invited the scientific community to explore the characteristics of nanotechnology (Bayda et al., 2020).

Nanotechnology has been an emerging research field based on the smallest scale manipulable manufacturing techniques of material that can be applied to a certain degree of will. The use of vital infrastructures such as biotechnology, genetic engineering, and other disciplines allowed nanometer-scale manipulation (Wong et al., 2013). The characteristics of novel biomaterials at the nanoscale have brought a powerful tool to achieve precise and smart functions, e.g., drug delivery, a localized effect dependent on size, a feature triggered by stimuli (Lombardo et al., 2019; Palestino et al., 2020).

The presented work explores the most prominent alternatives focused on modern medicine applied in tissue engineering, as well as therapeutics and vaccines for COVID-19 disease. First, a detailed description of nanostructures is given to narrow the kind of nanomaterials. Then, a description of the administrative mechanisms to understand conditions for the materials and characteristics of the structures used according to applications. The main topics for application are described as two of the new medicine targets in the global community to progress and impact. Tissue engineering can help a vast number of diseases, including the current COVID 19 pandemic by the angle of regenerative medicine. In general, multidimensional applications refer to the inclusion of several materials, drugs, geometries, and other characteristics in the nano biomaterial cues for desired multipurpose.

## Defining Nanostructures

Nanostructured materials (NMs) are a class of material that has at least one dimension on the nanometric scale (<100 nm) (Auffan et al., 2009). These are categorized according to the number of sizes that are not confined to the nanometric scale, being 0D, 1D, 2D, and 3D (Table 1; Tiwari et al., 2012). Their composition, in turn, groups them into metallic, semiconductor, ceramic, polymeric, carbon-based, and lipid-based. Due to its size in the nanoscale shape or structure, they display novel physical, chemical, and biological properties (Bhatia, 2016; Khan et al., 2019). The utilization of these materials is far-reaching, specifically in the biomedical field, being used as adjuvants in vaccines or for smart drug delivery (Fathi-Achachelouei et al., 2019).

**TABLE 1:** Nanomaterials classification is based on dimensions and their applications in biomedicine. Created with BioRender.com.

**Dimensions in nanoscale**	**Type of class**	**Schematic view**		**Biomedical applications**	**References**
0D	Nanoparticles	Nanospheres	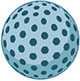	Tissue engineering and regenerative medicine- Gold: Minimize tumor recurrence, monitoring cancer relapse thru cell targeting, enhance cell differentiation, and wound healing applications.	Fathi-Achachelouei et al., 2019
		Nanoclusters	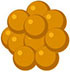	Silver: Prevent antimicrobial infections (wound healing). Ceramics: Enhancement of cellular activity, control of biomechanical properties, imaging, antimicrobial agents. Polymeric: Delivery of bioactive agents, imaging.	
1D	Nanofibers family	Nanofibers	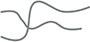	Polymeric: Drug delivery, antibacterial meshes, wound dressing, ECM mimicking for tissue engineering. Peptide: 3D cell culture, tissue repair, rapid hemostasis.	Dolez, 2015; Rezaei et al., 2016; Barhoum et al., 2019; Wu et al., 2020
		Nanowires and nanorods	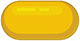	Gold: Photothermal and photodynamic therapy, contrast agent for imaging (laser optoacoustic, two-photon, photoacoustic and dual molecular imaging), biosensors, drug delivery. Calcium phosphates: Nanofillers for bone tissue engineering, drug delivery.	Liu et al., 2017; Sylvester et al., 2020
		Nanotubes	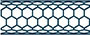	Carbon: Biosensors, contrast agents for imaging (MRI and NRI imaging), drug delivery (cancer and neurodegenerative diseases), and neuron scaffolds.	Wang et al., 2019
2D	Nanolayers	Thin films and nanoplates	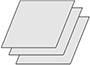	Metal oxides: Nanofillers for antibacterial films. Bioactive glass: Implant coating for improved bone mineralization.	Bauer et al., 2004; Ghassan et al., 2019
3D	Bulk nanomaterials	Nanocomposites	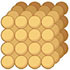	Inorganic: Contrast agents for MRI, magnetothermal-chemotherapy, biosensors, photothermal and photodynamic therapy, bright field detection. Polymeric: Scaffolds for tissue engineering, drug delivery, imaging, wound dressing, homeostatic agents, and biosensors.	Perez-Juste et al., 2005; Smith et al., 2009; Marangoni et al., 2016

In the wide range of nanostructures there are nanoparticles, nanofibers, nanorods, nanotubes, nanolayers and nanocomposites. The Nanoparticles (NPs), cataloged as 0-D nanomaterials, exhibit different morphologies, among them nanospheres, dendrimers, hollow spheres, cubes, rings, flowers, and micelles (Dolez, 2015). For the biomedical field, precise control of the delivery of bioactive agents is required, especially in tissue engineering, within a scaffold so that *in vivo* maturation can be successfully carried out. Throughout, recent focus has been given to responsive multifunctional systems instead of simple delivery systems to improve their drug load capacity and targeting even more. In addition, the use of nanoparticles systems in tissue engineering is based on their composition (Fathi-Achachelouei et al., 2019).

On the other hand, nanofibers, classified as 1D nanomaterial, have countless advantages such as the wide variety of materials with the possibility to transform into nanofibers structures high porosity, high proficiency of mechanical properties, significant ability to immobilize biological elements on the surface of the nanofiber (Rezaei et al., 2016). In the development of a new therapy based on nanofibers, penetration into cells, texture, composition, the molecular orientation of the nanofibers, and network structure must be regulated to improve its bioactivity (Barhoum et al., 2019). Electrospun nanofiber meshes derived from synthetic and natural polymers produce higher mechanical movements to boost the healing process. Natural polymers have added advantages such as biodegradability and antimicrobial properties. Meanwhile, synthetic polymers are better to formulate scaffolds combining crystallinity to ensure mechanical properties (Sylvester et al., 2020; Wu et al., 2020). Electrospun nanofibers are used to promote the rapid hemostasis process due to their high porosity and facilitate cell proliferation in wound healing (Liu et al., 2017). Also, they promote cell proliferation and differentiation applied in 3D cell culture and tissue repair (Wang et al., 2019).

Likewise, nanorods are 1-D nanomaterials and are composed of diverse materials as ceramics, metals, or carbon (Ghassan et al., 2019). Due to their aspect ratio, nanorods display a chemical, electrical, magnetic, and optical anisotropy, which allows a different interaction with biomolecules or cells (Bauer et al., 2004). Research has been focused mainly on gold and calcium phosphates nanorods. The first mentioned is primarily applied in photodynamic therapy and imaging (Marangoni et al., 2016). Moreover, nanorods exhibit an advantage over their spherical counterpart since they present two bands of surface plasmon resonance, where the longitudinal band absorbs in the near-infrared region where the maximum radiation penetration into the tissue occurs. Simultaneously, they have also been studied for drug delivery due to their ease of functionalization (Perez-Juste et al., 2005; Smith et al., 2009). Calcium phosphate nanorods, mainly hydroxyapatite, have been used both as nanocarriers and for bone tissue regeneration in composite scaffolds (Rubin et al., 2003; Nga et al., 2014; Dave et al., 2019; Li et al., 2019; Nakayama et al., 2019).

Nanotubes, in the same way, belong to the nanofibers family, having a diameter of a few tens of nanometers and extended length, but hollow. Carbon nanotubes (CNTs) and recently, halloysite nanotubes (HNTs) are predominantly studied. CNTs consist of layers of graphene rolled to form a cylinder, they can be single or multi-walled, and based on the carbon arrangement, can either be metallic or semiconducting (Rakhi, 2019). The nanotubes are studied in the biomedical field due to their ability to cross the cell membrane. Applications have an ample range, for instance, the production of biosensors, their use as a contrast agent in computed tomography (Negri et al., 2020), as nanocarriers (being the right candidate for DNA or RNA attachment) for its use in gene therapy (Kam et al., 2005), or neurodegenerative diseases therapy, since they can cross the blood-brain barrier (Kafa et al., 2016). When it comes to tissue engineering, carbon nanotubes can support and promote the proliferation of different tissues, mainly neural and cardiac cells. Recently, it also has been given attention for its use in stem cell culture, capable of modulating the proliferation and differentiation of diverse stem cells (Lee et al., 2015), e.g., mouse neural stem cells to neurons and oligodendrocytes (Jan and Kotov, 2007).

On the other hand, HNTs are composed of aluminosilicate layers (Du et al., 2010). Their surface chemistry stands out, being negatively charged on the outside and positively on the inside. This charge allows them to bind in their positive lumen synthetic and biological structures negatively charged, such as DNA (Rozhina et al., 2020). Furthermore, they are suited for drug delivery, tissue engineering, wound healing, and imaging, as they have high mechanical strength, excellent biocompatibility, and homeostasis properties (Satish et al., 2019).

Two-dimensional nanomaterials are classified as nanoplates, nanosheets, nano-disks, and nano-prisms. A thick graphene oxide nanosheet coated with gold nanoparticles has been used to identify cancer cell protein biomarkers (Ramanathan et al., 2019). Polymer-coated graphene oxide nanosheets have demonstrated biological properties against gram-positive and negative bacteria (Mahmoudi et al., 2016).

Finally, organic/inorganic hybrid nanocomposites are materials of nanoscale dimensions; the organic and inorganic materials determine the physicochemical, thermal, and mechanical properties, where porosity facilitates drug-delivery uses with applications as scaffolds for tissue regeneration encapsulating agents to trigger cell differentiation (Park et al., 2020). 3D constructs in tissue engineering are bioinspired in nanocomposites based on nano-hydroxyapatite (n-HAP) and poly lactic-co glycolic acid (Hassan et al., 2019), polylactic acid (PLA), and nHAP (Marycz et al., 2020), polyacrylonitrile-multiwalled carbon nanotubes (Samadian et al., 2020) and natural biopolymers such as alginate, chitosan, collagen, fibrin, and gelatin (Christy et al., 2020). The above description regarding nanostructures summarizes that the size, shape, and materials define the properties and hence the applicability, being biosensors, drug delivery, tissue engineering, and imaging predominantly studied. Ideally, nanoparticles possess the ability to create smart drug delivery systems. Nevertheless, other structures such as nanorods and nanotubes have been used as carriers of drugs. Most of the mentioned nanostructures have demonstrated their applicability in tissue engineering.

The examples mentioned before are just a summary of the wide range of nanostructures, which are cataloged as novel technologies for therapeutic purposes. This variety is due to the several factors involved to guarantee a correct delivery and successful effect of the drugs, considering all the physical, chemical, and biological barriers found in the human body. Besides the type of NMs and their applications, it is necessary to do an analysis of the delivery routes. Thus, to have a complete scenario of the implications of NMs for therapeutic purposes.

## Mechanisms of the Main Routes for Drug Delivery

Nowadays, nanotechnology is a fundamental component of modern medicine, specifically for drug delivery. Specifically, systems based on NMs have been considered perfect candidates for drug delivery. The main reason is due to their biocompatibility, high stability, and biodegradability. However, as with any scientific research, the development of those systems has brought complex challenges.

In this regard, before exploring the potential applications of NMs it is important to understand how NMs work to guarantee an efficient delivery and the anatomical mechanisms involved. Thus, the passive and active mechanisms have been implemented to locate the zone of interest, which can be an organ, tissue, or cell (Neves et al., 2016).

Passive targeting is a mechanism that can use either micro or nanoparticles. It works by accumulating the nano-drug onto the affected area, and its success depends on the durability of the NPs coating to remain in the bloodstream. This approach is mainly used for tumors, which is a disorganized structure with highly dilated vessels and big pores. This environment allows the migration of molecules up to 400 nm in diameter into the surrounding region. Thus, NPs can accumulate in tumor tissues and achieve a therapeutic effect (Brunaugh et al., 2019a).

On the other hand, active targeting uses mechanical and physical methods to enhance permeability. The objective is to attach the drug onto the surface of the site of interest, based on strong molecular interactions as a ligand-receptor (e.g., antigen-antibody), to deliver the drugs. The ligand responsible for this attachment is coupled within the NMs surface to facilitate the interaction with the receptor, targeting the specific site of action (Chenthamara et al., 2019).

**FIGURE 1:**
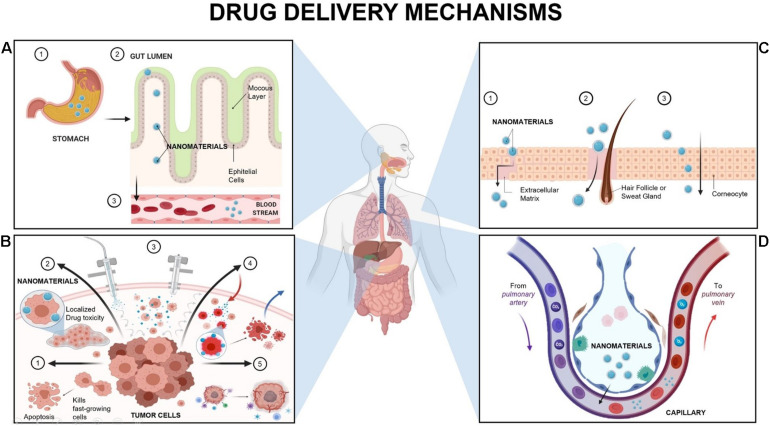
Principal Mechanism of Nanomaterials delivery in humans. **(A)** Oral delivery: The nanomaterial envelope plays the most crucial role as a protective agent. It allows the nanomaterials to pass from the stomach to the small intestine and reach the systemic circulation. **(B)** Intraperitoneal (IP) Delivery: The most common alternatives are Nanomaterials (NMs) loaded with chemotherapeutics [B1] to ensure the prevalence of the chemotherapeutics onto the target zone and induce apoptosis of fast-growing cancer cells (Dakwar et al., 2017). Secondly, depot systems for sustained release of nanomaterials (such as hydrogels) [B2], where the agents have localized toxicity (Van de Sande et al., 2020). Also, Pressurized intraperitoneal aerosol chemotherapy (PIPAC) [B3], in which nanomaterials are released as close to the tumor as possible, and then extracted to avoid healthy cells damage; followed by Hyperthermic IP chemoperfusion (HIPEC) [B4], which uses heated chemotherapy (107 degrees) with NMs to reduce cancer recurrence (Carlier et al., 2017; Alyami et al., 2019; Van de Sande et al., 2020). Finally, Metronomic therapy of NMs [B5], which induces the innate immune response and inhibits tumor angiogenesis (Carlier et al., 2017; Dakwar et al., 2017; Kamble et al., 2017; Alyami et al., 2019; Van de Sande et al., 2020). **(C)** Skin delivery: It has three alternatives to go through the stratum corneum, which are the intercellular route [C1], the appendageal route [C2], or the transcellular route [C3]. The intercellular route means to pass around the corneocytes, using the hair follicles as a pathway for drug permeation, specifically for particles around 600 μm (Sala et al., 2018; Brunaugh et al., 2019c). On the other hand, the appendageal route is preferably for hydrophobic or high molecular-weight NMs (Brunaugh et al., 2019c). In this route, the NMs passes through the epidermis to the dermis using aqueous microchannels formed around the hair follicles, sebaceous glands, and sweat glands (Patel et al., 2018; Zhou et al., 2018). In the transcellular route, the molecule passes through the corneocytes (phospholipid membrane and cytoplasm) and lipid lamellae; and it is commonly used for polar molecules (Sala et al., 2018; Carter et al., 2019; Kim et al., 2019). **(D)** Gas exchange delivery: Due to the size of NMs, the vast majority pass through the upper region of the airways (Paranjpe and Müller-Goymann, 2014). Once in the alveoli, nanocarriers will find tight junctions between the epithelial cells (alveolar primary barrier), and a set of proteins and lipids (known as alveolar lining). At this point, the physicochemical properties of NMs determines if it passes via active absorption or passive diffusion. In addition, the nanocarrier should be able to resist the degradation activity of enzymes such as cytochrome P450. Finally, the NMs can be taken up either by the alveolar surface cells (where are further absorbed into the systemic circulation) or can be phagocytized by the alveolar macrophages (Chenthamara et al., 2019). Created with BioRender.com.

Both mechanisms are currently implemented for drug delivery; nonetheless, the route of delivery would be the decisive factor in choosing the most suitable arrangement (Chenthamara et al., 2019). Several routes implemented for drug delivery exist; however, the most explored, based on nanotechnology, are described in the following subsections along with a schematic representation of the pathways and mechanisms presented in Figure 1.

### Oral

Oral drug delivery (ODD) is by far the preferred route of drug administration. Some of the advantages of this type of transportation are pain avoidance, efficacy, and risk reduction regarding infections due to the avoidance of needles (Neves et al., 2016). Nanocarriers, in general, have allowed oral delivery of hydrophobic or poorly water-soluble compounds, as well as targeting in difficult zones like the gastrointestinal tract (GIT). Thereby facilitating transportation across the gastrointestinal (GI) barrier, keeping the pharmaceutical properties of the drug, and increasing the absorption rate (Brunaugh et al., 2019a; Chenthamara et al., 2019).

The general mechanism (Figure 1A) starts with the intake of the drug. Later on, the nanocarriers are going to enter the GIT, which is divided into the stomach, small intestine (Duodenum, Jejunum, and Ileum), and the large intestine (He et al., 2019). This step is crucial for drug absorption due to the multiple layers presented in the GIT epithelium (mucosa, submucosa, muscularis externa, and the serosa) and the different types of cells (Nabi et al., 2019; Xu et al., 2020). The GIT starts with the stomach, where the drug is predigested by gastric acid and gastric lipases (Nabi et al., 2019). The gastric retention of the drug favors bioavailability and solubility, as well as reduces drug wastage (Nagendran, 2016). The next step is focused on targeting the affected area, meaning that the NMs can pass from the stomach to the small intestine by mechanical blending, or they may end up taken by the GIT cells, depending on the delivery target zone.

Another alternative (route known as transcytosis) (Zhang and Merlin, 2018) occurs not just in the stomach but in the whole GIT. Once the affected area is detected, the transcytosis starts with the endocytosis at the cell apical membrane. Moreover, NMs pass through the cells and are delivered to the basolateral pole. At this point, in the submucosal layer, NMs could interact with immune cells before they reach the systemic circulation or the targeting zone, where mainly inflammation is presented (Zhang and Merlin, 2018). For an efficient ODD, it is required to overcome all the physical barriers and biological components (as microbiota and enzymes), even before having access to the several types of GIT cells (Chenthamara et al., 2019). However, such unique variations combined with current advanced technology can be exploited to design increasingly specific ODD systems (Sardo et al., 2019).

### Intraperitoneal

In comparison to the others, the intraperitoneal route is only used in extreme cases due to the highly invasive steps involved during the treatment (Colby et al., 2017). Currently, it is considered as a potential alternative in the oncology field to treat Peritoneal Metastasis (PM), which is the phase where tumor cells originated in the gastrointestinal or gynecological tract, spread through the peritoneal cavity. In most cases, the early stages of this cancer go unnoticed, and when PM is diagnosed, the catalog of treatment options is minimal (Reymond and Königsrainer, 2020).

Under normal conditions, the peritoneal cavity is mainly a membrane (also known as a peritoneal-plasma barrier), composed of diverse layers of connective tissue with an average surface area of 1.5 m^2^, that covers visceral, abdominal, and pelvic organs (Dakwar et al., 2017). The first layer works as a barrier defense. It consists of mesothelial cells interconnected by tight junctions and coated by glycocalyx (a highly hydrated fibrous meshwork of carbohydrates) (Alyami et al., 2019). Then, it is followed by the sub mesothelial basement and the interstitial space, which contains collagen, fibroblast, and other components to protect the area against macromolecules. Lastly, a layer composed of negatively charged endothelial cells with the same purpose: avoid the entrance of macromolecules to the cavity (Carlier et al., 2017; Dakwar et al., 2017). However, this structure changes abruptly with the arrival of cancer cells due to the metastatic cascade. The cancer cells use the peritoneal fluid to spread rapidly, and the adhesion can occur in any layer. The oncotic pressure starts once the peritoneal microvessels become hyperpermeable, and with the secretion of pro-inflammatory cytokines and chemokines. On the other hand, tumor cells induce apoptosis of healthy cells, altering the peritoneal membrane structure (Van de Sande et al., 2020). Thus, due to the aggressiveness of this tumor and the low efficacy of current therapies, the most common therapeutic approach to treat PM consists of palliative systemic chemotherapy to prolong survival and ease symptoms but not cure disease (Reymond and Königsrainer, 2020).

According to the tumor nature, the key for treatment success lies in the drug capacity to accumulate in the affected area and the delayed clearance caused by the peritoneal plasma barrier (Van de Sande et al., 2020). A combination of dose intensification and frequency can maximize therapeutic effectiveness and minimizes side effects on the patient (Colby et al., 2017). Here is where nanostructures play a crucial role in developing therapies that guarantee the elimination of these tumors. Currently, several strategies under development proposed an approach based on NMs and local-regional treatment, where the most promising therapies for efficient IP drug delivery (Figure 1B; Van de Sande et al., 2020).

As an overview, intraperitoneal (IP) therapy is a growing niche to treat PM, but there are still several obstacles to overcome, despite the intensive effort of clinicians, pharmacologists, and material scientists, to reach a fully developed IP therapy based on nanomedicine. Therefore, to unravel the potential of NM-based IP therapies, further investigations should focus on two principles: Improve the biodistribution of nanomedicines in the peritoneum, and the correlation between biodistribution with tumor accumulation, penetration, and killing efficacy, to accelerate the development of these promising alternatives.

### Skin

The human skin is known as the largest organ in the human body, covering 16% of the total body surface area (1.8 to 2.0 m^2^) (Kamble et al., 2017). This organ is divided into three main layers. The epidermis, composed of multiple flattened cells over each other that lies onto the stratum Basale. This base layer is formed of columnar cells arranged perpendicularly as melanocytes and keratinocytes (Monteiro-Riviere and Riviere, 2009; Makhmalzade and Chavoshy, 2018). As a whole, besides minimization of water loss, the principal role of the skin is to act as a defense barrier, owing to prevent the invasion of foreign agents as organisms (virus, bacteria, fungi), dust, allergens, toxins, and particulate materials (Jijie et al., 2017). This crucial characteristic makes it a challenge for drug permeation and delivery, primarily due to the *stratum corneum* (SC), which confers the remarkable barrier properties of the skin (Brunaugh et al., 2019c).

Thus, the primary objective of NMs is to overcome this barrier to reach the bloodstream (Chenthamara et al., 2019). The ability of a drug to pass across the skin to be absorbed through the skin layers and exert a systemic effect is known as transdermal drug delivery (TDD) (Zhou et al., 2018). The effectiveness of this method is influenced by the drug concentration gradient, the partition coefficient, diffusion coefficient of the NM, and length of the pathway through the skin (Brunaugh et al., 2019c). Based on passive diffusion, NMs can pass into the skin to reach the desired target by two main routes: passing through the trans-epidermal path (SC) or via the appendages. For the SC route, the molecule can penetrate either by the transcellular way or by the intercellular route (Patel et al., 2018; Chenthamara et al., 2019; Figure 1C).

Skin treatments based on nanotechnology have brought a wide range of new alternatives to treat skin diseases, just as psoriasis, alopecia, dermatitis, acne vulgaris, vitiligo, and even skin cancer (Sala et al., 2018). The implementation of nanomaterials has helped to guarantee an efficient delivery based on the protection of the drug mainly. Hence, the side effects have been reduced, improving patient acceptance. On the other hand, the skin route has opened the opportunity to face different types of diseases, such as diabetes, Parkinson’s, Alzheimer’s, osteoporosis, among others (Carter et al., 2019; Kim et al., 2019).

Currently, drug delivery based on skin penetration has shown promising results regarding therapies based on NMs; However, there is still a lot of improvement to reach an efficient distribution of macroparticles (as genes, proteins, and drugs) (Rabiei et al., 2020). Therefore, nano-formulations can work in combination with other molecular techniques as nanoneedles or nano patches to eliminate deficiencies associated with penetration or long-term stability of the drug, bringing new approaches for drug delivery.

### Gas Exchange Regions

The term gas exchange refers to the delivery of oxygen (O_2_) and the elimination of carbon dioxide (CO_2_). The O_2_ is delivered from the lungs to the bloodstream. Meanwhile, the CO_2_ is released by following the opposite direction. This vital process is known as respiration, and it occurs in the lungs, more specifically, in the alveoli (Paranjpe and Müller-Goymann, 2014). The lungs are composed of two functional parts: the airways (trachea, bronchi, and bronchioles), numerous bifurcations that get narrower and shorter inside the lungs (Karra et al., 2019), and the 300 million microscopic air sacs known as alveoli. Besides respiratory organs, the lungs also have lymph tissue, and the alveoli are lined with over 280 billion capillaries. This network is known as the blood-gas barrier, where the distance between an alveolus and a capillary is just about 0.5μ*m*, allowing the gas exchange by diffusion (Paranjpe and Müller-Goymann, 2014).

The presentations available for NMs delivery through the inhalation route (IR) are pressurized metered-dose inhalers, nebulizers, and dry powder inhalers, and the most commonly used NM are those with a sphere configuration, such as nanoparticles (NPs) (Chenthamara et al., 2019). The NM measuring less than 20 nm is delivered to the alveoli, and often they present low retention, mainly due to the rapid penetration into the bloodstream (Sankhe et al., 2019; Thakur et al., 2020). The most common mechanisms for NM delivery are sedimentation and diffusion (also known as Brownian motion) (Labiris and Dolovich, 2003). The first one occurs due to gravitational forces but is also influenced by the breathing pattern, where slow breathing provides sufficient time for sedimentation (Chellappan et al., 2020). This mechanism allows NM to settle for a long time in the smaller airways and bronchioles, increasing the efficiency of the drug. On the other hand, in the case of diffusion, the particles smaller than 0.5 μm end up directly in the alveolar region where the air flow rate is low (Brunaugh et al., 2019b). Thus, it strongly depends on drug dissolution in the alveolar fluid, the concentration gradient, and contact with the lung surface (Thakur et al., 2020).

Sedimentation is the most attractive method for NMs systems; however, diffusion is also really used for those systems focused on reaching the circulatory system rapidly and efficiently (Paranjpe and Müller-Goymann, 2014).

Regardless of the chosen delivery mechanism, there are several barriers to overcome before entering in contact with the alveolar region (physical, chemical, and immunological) to preserve homeostasis (Karra et al., 2019; Figure 1D).

Drug delivery systems suitable for IR development must consider some characteristics of the particles such as density, charge, shape and diameter, solubility, and hygroscopicity. Moreover, certain factors regarding the patient’s respiratory system as airflow velocity and airway structure (Pannonhalminé Csóka et al., 2019). However, despite the complexity of this pathway, the IR is attractive for several pulmonary diseases, where the speed reaction and focal delivery are crucial. That is the case of asthma, chronic obstructive pulmonary disease (COPD), cystic fibrosis, pulmonary hypertension, and infections like tuberculosis, pneumonia or the current pandemic COVID-19 (Mehta et al., 2019).

There are main advantages of drug delivery to gas exchange regions over other routes as ODD or injection besides being a noninvasive system. These are the evasion of hepatic metabolism and GIT; the rapid onset of therapeutic effect due to direct delivery; lower dose requirement and rapid absorption because of high vascularization; more-concentrated drug distributed to the site of action; and above all, a thin barrier to cross to get through the systemic circulation (Ho et al., 2019).

There are preferable routes used, such as the oral delivery and respiratory region for drug delivery; however, as technology and medicine advance, more alternative pathways are considered to improve drug efficiency. One clear example is the intraperitoneal route, which has shown promising results in combination with NMs due to the direct application towards the tumors. The powerful combination of NMs and current drugs brings us a broad spectrum of possibilities to personalize treatments, not only according to the patient but to the disease itself, increasing the rate of success and guaranteeing a better life quality, as the modern examples mention in the following section.

## Modern World Applications

### COVID-19: Current Challenges and the Therapeutic Role of Multidimensional Nanostructured Materials

The COVID-19 disease is caused by the SARS-CoV-2 virus, which compromises the respiratory system by causing an acute immunological response. Ultimately this leads to death with a fatality rate per country ranging 0.05-19.4% for extreme cases, according to the Johns Hopkins Coronavirus Resource Center. The SARS-CoV-2 affects the respiratory system by preventing the correct oxygenation of blood as a result of increased mucous secretions that clog alveoli as well as tissue inflammation (Ullah et al., 2020). It also infects alveoli at the lungs by its endocytosis and replication, which generates an acute immune response (Glebov, 2020). Furthermore, it triggers the signal cascade for an acute inflammatory response through cytokine storms (Ullah et al., 2020). In addition to the initial attack on the respiratory system, the virus spreads to the digestive system, mainly affecting the colon, distal kidney, olfactory nervous, pancreas, liver, and potentially every tissue that expresses ACE2 receptor (Gavriatopoulou et al., 2020). The academic paradigm of COVID-19 pathology is thus shifting from a respiratory-only focus into a systemic syndrome with long-term damage of affected organs (Gavriatopoulou et al., 2020).

**FIGURE 2:**
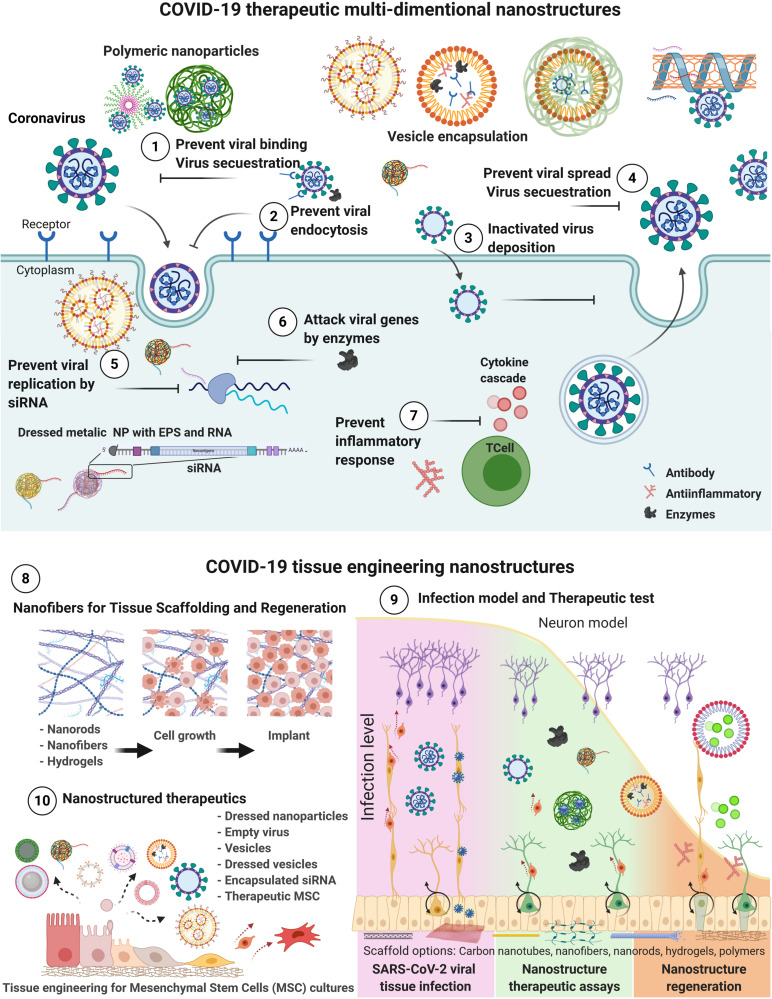
Schematic representation of nanostructured-based technology focused to COVID-19 therapeutics. Therapeutic action mechanisms for COVID-19 treatments by drug delivery, dressed nanoparticles, vesicles carriers and MSC targeting different infection processes from (1 to 7). Tissue engineering nanostructured-based technology for (8) tissue regeneration, and implant preparation; (9) test infection, generate therapeutic assays, regeneration; and (10) Nano therapeutics production of vesicles, nanoparticles, and MSCs (Basu et al., 2020; Gordon et al., 2020; Gupta S. et al., 2020; Hassanzadeh, 2020; Hu et al., 2020; Inal, 2020; Leng et al., 2020; Lin et al., 2020; Mohammadi et al., 2020; Moon et al., 2020; Muralidharan et al., 2020; O’Driscoll, 2020; Petit et al., 2020; Yu et al., 2020; Zhang et al., 2020). Created with BioRender.com.

The lack of adequate policies for the containment of COVID-19 infections has caused an international public health emergency with economic implications, as the only proven mechanism to halt its spread is social distancing (Vabret et al., 2020). The current pandemic thus demands scientific solutions for the prevention, treatment, and containment of the disease to minimize further infections and deaths at a global scale. Nanotechnology provides opportunities to tackle COVID-19 infection from four different approaches; point of care (POC) diagnostics, surveillance and monitoring, therapeutics, and vaccine development (Chan, 2020). Additionally, tissue engineering based on nanotechnology serves as a complementary tool that allows the *in-vitro* assessment of therapeutic strategies, generation of bio-nanostructures that act as scaffolds for the regenerative treatment of affected patients, and development of *in vitro* tissue models for the research of the extensive effects of COVID-19 in different organs (Shpichka et al., 2020). This subsection highlights the current developments in the fields of nanotechnology and tissue engineering with potential applications for therapeutics, vaccine carrying, tissue replacement, immunomodulation, and smart drug delivery to treat this disease. An overview of the presented strategies and the multidimensional applications of nanotechnology for COVID-19 is presented in Figure 2.

### Tissue Engineering for Immunomodulation and Replacement of Tissues Damaged by COVID-19

Over the past two decades, combined progress on the nanomaterial and stem cell fields has led to the establishment of tissue engineering as a prolific ground for the research of organs *in vitro* and the progress of regenerative therapies (Hoffman et al., 2019), which has potential applications on COVID-19 therapeutics. The adaptation of *in vitro* studies into clinical trials demands the advancement of tissue models, through the development of scaffolds with a high level of vascularization, intricate cell signaling, and complex matrix structure (Khademhosseini and Langer, 2016). COVID-19 promotes inflammation of several tissues (Gavriatopoulou et al., 2020), which damage their integrity, so desirable features of synthetic tissue scaffolds must be included. For instance, an acceptable toxicity profile, high biocompatibility, mechanical properties that replicate the implantation tissue, and biodegradability to ensure scaffold removal without the need for invasive surgery (Teixeira et al., 2020).

Over the past two decades, combined progress on the nanomaterial and stem cell fields has led to the establishment of tissue engineering as a prolific ground for the research of organs *in vitro* and the advancement of regenerative therapies (Hoffman et al., 2019), which has potential applications on COVID-19 therapeutics. The adaptation of *in vitro* studies into clinical trials demands the improvement of tissue models through the development of scaffolds with a high level of vascularization, intricate cell signaling, and complex matrix structure (Khademhosseini and Langer, 2016). COVID-19 promotes inflammation of several tissues (Gavriatopoulou et al., 2020), which damages their integrity. Therefore, it is desirable for synthetic tissue scaffolds to display an acceptable toxicity profile, high biocompatibility, mechanical properties that replicate the implantation tissue, and biodegradability to ensure scaffold removal without the need for invasive surgery (Teixeira et al., 2020).

Nanofibers have become the staple nanostructure for tissue engineering scaffolds, owing to their large surface area to volume ratio, high surface modifiability, three-dimensional flexibility, and high tensile strength (Parham et al., 2020). Electrospinning has gained widespread adoption among available methods of nanofiber fabrication for the manufacture of tissue scaffolds, owing to its high adaptability under many process requirements and environmental factors (Alavarse et al., 2017). Important scaffold morphology parameters like pore size distribution and surface area to volume ratio can be easily adjusted through this technique (Felgueiras et al., 2019). The combination of topographic and biochemical modification of nanofibers that electrospinning offers makes accurate emulation of the extracellular matrix possible, enabling the adhesion and proliferation of cells over extended periods (Wang et al., 2013; Teixeira et al., 2020). Natural polymers like collagen, alginate, and chitosan are preferred for nanofiber scaffolds due to overall better cytocompatibility and synaptic plasticity, drug compatibility, biodegradability, due to COVID-19 urgency the regulation institutions can provide fast track to this type of materials. However, synthetic biomaterials can provide better technical flexibility in specialized situations (Dhasmana and Zzaman, 2019). Popular choices for synthetic polymers include poly-(lactic acid), poly-(glycolic acid), and poly-(ε-caprolactone), as these materials have good mechanical stability and conductivity, albeit lack bioactivity (Álvarez-Suárez et al., 2020), some of the capacities required in COVID-19 disease damages to blood vessels, pulmonary tissue, heart valves, brain-blood barrier, neurons, and other cells, epithelia, tissue and organs (Gavriatopoulou et al., 2020).

The surface of electrospun polymer fibers can be modified through the addition of functional groups and bioactive compounds like hormones and cytokines to cue cell signaling and immunomodulation (Li et al., 2004; Ulery et al., 2011), where it may play a crucial role to prevent the damage caused by the immune response to SARS-CoV-2 infection on alveoli and other affected tissues (Çetin and Topçul, 2020). Most notably, genetic material can be loaded into the nanofiber scaffold to guide stem cells through a highly specific differentiation profile (Park et al., 2016). The scaffold applied in the regeneration of damaged tissues by COVID-19, such as angiogenesis to tackle stroke dame, neuron regeneration, or modulate inflammation. The encapsulation of genes and signaling molecules into the scaffold also provides better control for the delivery of molecules over a prolonged time (Gonzalez-Fernandez et al., 2016). The overall result of these chemical enrichment strategies are scaffolds that are highly applicable for the regeneration and replacement of wounded human tissues, that are also applicable to the modern problem of COVID-19 therapeutics, and that already have been applied with success on several organ systems (Hassanzadeh, 2020).

Given the severe onset of COVID-19 related tissue damage at a systemic level in extreme cases, the application of stem cell engineering to repair and regain organ functionality has been explored and adapted from other applications to quickly answer the need for potential therapeutic solutions (Ji et al., 2020). The usage of nano scaffolds based on biocompatible polymers for tissue engineering using mesenchymal stem cells (MSCs) has thus emerged in articles as a promising experimental therapeutic option for patients at a critical stage to reduce inflammation, enhance immunomodulation and promote tissue repair back to a functional level (Esquivel et al., 2020). A summary of the potential uses of this technology is briefly discussed.

The usage of MSCs and the modulation of its expression and immune response through trophic factors and cytokines is an appealing prospect to reduce and repair lung tissue injury on the onset of an inflammatory infection such as that caused by SARS-CoV-2 (Li et al., 2020). Stem cell therapy has been proposed as a complementary treatment parallel to antiviral drugs for influenza in cases with severe pneumonia (Çetin and Topçul, 2020). This approach has been reported and used to treat H7N9-ARDS in a study (Chen et al., 2020), where the transplantation of MSCs derived from menstrual blood reduced inflammation and the onset of cytokine cascades, improving lung function without any short-term side effects. The authors note that the similarities of the acute respiratory syndrome caused by SARS-CoV-2 and the H7N9 influenza virus may allow this strategy to be applied to the ongoing pandemic to reduce the mortality of most severe cases.

Along with the immunomodulatory effect of stem cells on lung tissue, the complete replacement of injured lung structures for artificial structures that incorporate tissue engineering has also been proposed as an alternative to alleviate the demand for donated organs (Swol et al., 2020). Under this concept, bio-fabricated scaffolds with microfluidic channels enable a high level of biocompatibility and reduced instrumentation sizes, as well as device development flexibility through the adaptation of mechanical components for ventilation. However, the quick and complete differentiation of stem cells minimizes the reproducibility of potential commercial products and hinders biosafety assay efforts, limiting the implementation of developed bio-artificial lungs on clinical trials (Li et al., 2020).

The expression of ACE2 in neurons and glial cells makes them a potential target of SARS-CoV-2 with the onset of neurological symptoms like numbness and chronic pain. The infection may also cause neuromuscular disorders due to prolonged damage to the brain and spinal cord structures (Ftiha et al., 2020). The coagulopathy associated with COVID-19 brings secondary damage to neural structures through stroke events (Kipshidze et al., 2020). Given the lack of antiviral agents with a potent effect over a severe COVID-19 infection, the regeneration of neural structures post-infection can provide an attractive treatment to regain damaged tissue. The *in vitro* culturing of neural cells has been posed as a potential therapy for neurodegenerative diseases through cell replacement (Dhasmana and Zzaman, 2019), where conventional neural culturing agents like Matrigel have proved unsuccessful due to inadequately emulating the highly specific requirements of the perineuronal net (Wang and Fawcett, 2012). Neurons cultured in Matrigel lack guided directionality, develop glial scars, and have lower rates of migration (Ma et al., 2008; Moeendarbary et al., 2017) compared to electrospun scaffolds. A work by Cerrone et al. (2020) shows that electrospun scaffolds promote longer growth and directionality of dendrites with lower rates of apoptosis. Further studies are required to develop optimal blends of biopolymers for each application, as well as to measure the viability of in-vivo implantation and migration from the scaffold to the damaged tissue to show the full potential of electrospun nano-scaffolds for tissue regeneration (Cerrone et al., 2020).

Alternatives to transplantation are actively being proposed as a solution to overcome the shortage of available resources. One such approach is the use of synthetic scaffolds as a framework for cell proliferation to substitute damaged organs with functionally similar synthetic tissues. As with other body structures, ACE2 receptors can be found on the surface of corneal tissue, and potential complications of the COVID-19 infection may result in corneal ulcers and permanent damage, requiring transplant (Gupta P.C. et al., 2020). Nevertheless, current health guidelines advise avoiding the donation of corneal tissue on those patients recently (28 days) infected, as the eye may be a potential access point for infection (Eye Bank Association of America, 2020). Corneal transplant restrictions reveal an emerging issue for tissue donation, exacerbating the shortage of available donors and resulting in waiting lists for affected patients (Tan et al., 2012; Gain et al., 2016). Synthetic corneal scaffolds need acceptable transparency and mechanical requirements that cannot be met by conventional techniques and available materials (Ahearne et al., 2009). Electrospun fiber scaffolds have recently been developed as a potential replacement to traditional corneal substitutes such as amniotic membrane transplantation (Fernández-Pérez et al., 2020; Hasbiyani et al., 2020). The main benefits that they pose compared to conventional approaches are lower risks of contamination and higher compatibility of donor and receptor tissues. As with cornea, cells on other frequently transplanted organs may express the ACE2 receptor that makes them targets for SARS-CoV-2 tissular damage, making the development of synthetic grafts crucial as a potential therapeutic tool (Gupta S. et al., 2020). Scaffolds have also been developed successfully for organs with a higher level of physiological complexity, such as the liver (Grant et al., 2019) and pancreas’ islets of Langerhans (Buitinga et al., 2013). Electrospun scaffolds also show promise for the high-scale fabrication of vascular grafts to repair damaged cardiovascular arteries (Hasan et al., 2014). Their implantation into human patients remains a challenge, though, as the limited emulation of mechanical and cell attachment properties limits their long-term usability.

The shortcomings of electrospun fibers like fragility and solvent cytotoxicity can be overcome through the usage of emerging technologies for scaffold production. The combination of nanomaterials with 3D printing opens a new field of potential regenerative therapy models at a low cost (Di Marzio et al., 2020). 3D bioprinting (3DBP) is the automated manufacture of biological constructs from base materials with high control over geometry, material composition, and cellular distribution (Pedde et al., 2017). Compared to electrospinning, 3DBP offers scaffolds with better mechanical capabilities thanks to a careful geometric layout that enable their use on hard use tissues such as the heart (Yang et al., 2011), another potential target of COVID-19 infection through inflammatory and necrotic effects (Gavriatopoulou et al., 2020).

Processes for material deposition include jetting, sintering, extrusion, and stereolithography (Papaioannou et al., 2019). Each technique has advantages and shortcomings on properties like printing resolution, biocompatibility that influence the final product. Droplet size can vary from 300 μm for inkjet printing to >50 μm for stereolithography, and the viability of cultured cells can range from >95% for laser-assisted printing to as low as 25% for stereolithography (Melchiorri et al., 2016). The biological, structural, and economical requirements of the scaffold must also be considered when choosing the pairing of bio-ink composition and bioprinting technique (Pedde et al., 2017). Good ‘printability’ properties (e.g., shear thinning) must also be considered and are dependent on the used 3DBP process (Di Marzio et al., 2020). Thermogels like Pluronic (poloxamer) have been notably used for 3D bioprinting applications. However, a high concentration of this material is detrimental to cell viability (Fedorovich et al., 2009). Pluronic-based bioinks usually have a high concentration at the beginning steps of printing and remove most of it once cell culture starts (Müller et al., 2015). Besides Pluronic bioinks, natural polymer-based materials like cellulose and chitosan have been used to enhance cytotoxicity responses (Nguyen et al., 2017).

As with electrospun scaffolds, bio-printed tissue grafts have been fabricated as a potential solution to the demand for transplantable organs or the replacement of damaged tissue structures affected by severe COVID-19. Hydrogels have been used to 3D bio-print frameworks for organs like the liver, where a study successfully cultured hepatocytes on collagen and chitosan scaffolds to generate a synthetic organ that was engrafted to mice (Zhong et al., 2016). Examination after two weeks showed that viability was minimally impacted by the 3D scaffold. Advanced 3D bio-printed models for the heart also exist and range in complexity from capillary to full-organ development (Lee et al., 2019). Integration with induced pluripotent stem cells, as well as their differentiation into the full tissue’s cellular environment, is important to ensure graft viability after transplantation, as implant procedures for heart grafts in mice show (Maiullari et al., 2018). Other successful recent models of 3D bioprinted organs that may prove interesting for COVID-19 treatment include nerve (Liu et al., 2020), trachea (Kim et al., 2020), and lung (Galliger et al., 2019), yet many more exist for this emergent topic. It is important to note that the large-scale clinical implementation of bio-printed scaffolds is still limited due to ethical and technical concerns (Kirillova et al., 2020), and most studies restrict their application to animal models that cannot fully replicate human conditions (Ravnic et al., 2017). Thus, the true impact of bio-printed scaffolds on regenerative medicine remains to be seen.

Tissue engineering may benefit from the development of remotely controlled bioinks that change conformation through different stimuli thanks to the addition of doping agents like metal nanoparticles (Gao et al., 2016). A structure that may be adequate for cell proliferation can be subsequently changed once the tissue matures without an external, direct force for its functionalization (Di Marzio et al., 2020). These changes of structural conformation over time have been labeled as 4D bioprinting, and recent reports have shown the array of potential applications that smart nanomaterials can provide. Stimulation of bio-printed scaffolds has been used for the fabrication of structures with functionality like bone grafts, where 4D strategies confer better microvasculature compared to static scaffolds (Barabaschi et al., 2015). 4D bioprinting has also enabled the study and replication of cortical folding, furtherly elucidating axonal growth and neural maturation that enhance the next generation of neural tissue models (Miao et al., 2018). Smart structures created through 4D bioprinting may also act as a support for existing biological structures for therapy purposes, as conveyed by intravenous stents with shape memory capabilities (Ge et al., 2016).

As the integration of bioprinting and nanomaterials advances, tissues with higher complexity and functionality are expected to advance to degrees each time closer to complete organ replicas. Going forward, the main challenges for tissue engineering applied in the COVID-19 pandemic are the translation of clinical research into therapies and the development of scalable manufacturing strategies at a commercially viable measure (Hoffman et al., 2019). 3D printing shows promise at the reproducible, low-cost, and high throughput of scaffolds for tissue production, but the timespan for tissue maturation and cell survival must be optimized to ensure appropriate clinical intervention. Biosafety and acceptance by regulatory agencies are also a concern as there is a wide variety of nanomaterials used for trials (Gilbert et al., 2018). The integration of iPSCs, gene modification through CRISPR-Cas9, and the controlled release of bioactive compounds through specialized nanomaterials for directed cell differentiation opens the gate for personalized transplants, potentially hastening the approval and usage of future regenerative therapies while lowering demand for organ transplants (Pulgarin, 2017).

## Perspectives, Challenges, and Recommendations

In only a few months, SARS-CoV-2 has created a worldwide contingency mainly because of its rapid adaptation as an infecting agent throughout mutation. Even though viruses mutate constantly, the complication is that the virus acquired the ability to mutate in new variants with a selective advantage over the predecessor. These assets can induce higher viral loads, the ability to infect younger hosts, or more capable artillery to evade the immune system and go unnoticed. Recently, new variants were found in England (known as B.1.1.7 or VUI 202012/0), Brazil (named as P.1 or VOC202101/02 in the United Kingdom), and South Africa (known as 501Y.V2) (Mahase, 2021). As a result, the development of an efficient control has been challenging. Despite all the alternatives shown from 2020 to the present, currently, treatments that have proven to have a real effect in controlling COVID-19 symptoms are Dexamethasone, Remdesivir, Baricitinib in combination with Remdesivir, and Anticoagulation drugs (as heparin or enoxaparin) (Lammers et al., 2020). Other alternatives to treat patients have been convalescent plasma from people who have recovered from COVID-19; Monoclonal antibodies as bamlanivimab (LY-CoV555) and REGN-COV2 developed from the companies Eli Lilly and Regeneron, respectively, and AZD7442 from AstraZeneca (Mahmood et al., 2021). The novel panorama of variants impulses the necessity for specialized tools to test the effects of infection, immune system response, and therapeutic use of drugs and vaccines. One of those tools can be the tissue engineering applied to COVID-19 as *in vitro* test, vaccine production model, and tissue regeneration.

Regarding vaccines, 68 potential COVID-19 candidates are being tested in human clinical trials; however, the mRNA vaccines such as the one developed by Moderna, which is a prefusion of stabilized S protein, or the lipid nanoparticle mRNA vaccine developed by BioNTech, Pfizer, Fosun Pharma, the viral vector vaccines (as the Chimpanzee adenovirus vaccine vector ChAdOx1) by AstraZeneca and Oxford, and the adenoviral-based Russian Vaccine Sputnik V are leading as they have been already approved for emergency use in EEUU, UK and successfully approved in Canada. Also, they are the ones that are being distributed and applied worldwide. The main advantage of mRNA platforms is that it is non-integrating, posing no risk of insertional mutagenesis (Shin et al., 2020). In contrast, there is a lack of precise viral vector vaccines, yet their main assets are broad tissue tropism, inherent adjuvant qualities, and scalability. The pre-existing human immune response against those viruses complicates their efficiency. Thus, nanotechnology can be used to develop protein nanoparticles from antigenic subunits, or it can improve immunogenicity by using proteinaceous biomaterial scaffolds as ferritin and encapsulating (Shin et al., 2020). However, delivery is still a challenge, hence the nanotechnology platforms have been crucial to reaching their targets as these approaches can offer a solution. These being the use of cationic liposomes, polymeric nanoparticles, cationic nanoemulsions, liposomes, or dendrimers to ensure successful delivery through the cell membranes (Talebian et al., 2020).

Besides, this pandemic also made clear the need to cover other diseases due to their incidence on the world population. Some examples are cancer, cardiovascular diseases, respiratory syndromes, diabetes, and obesity, to mention a few. Herein, nanotechnology can potentially solve complex problems in a wide range of research fields, more specific to medical applications. The main idea of this review is to indicate that the multidimensional nanostructures exponentially increase the impact of medicine. For instance, the use of several molecules in tissue engineering and COVID-19 therapeutics brings the advantages of multipurpose treatment. Moreover, to achieve personalized medicine, the incorporation of smart materials by nanotechnology is necessary to improve tunable drug release profile, structural properties, prolonged effects, better biodegradation, specificity and biodistribution, reduce/eliminate toxicity, and side effects. Nonetheless, there is still research to be done for nanomaterials in the medical field (such as cytotoxicity and accumulation of nanoparticles or early-stage regulatory guidelines, which are opportunities for mid and long-term research) that limit their applicability at the present. With this revision, it is evident that options available now offered by nanotechnology and tissue engineering to fight against the COVID-19 pandemic are needed for therapeutics, drugs, and vaccine development. The applications will ensure efficient alternatives for the whole population as either medication, personalized therapies, or preventive treatments. In turn, it will strengthen our medical capacities and portfolio for this and future pandemics.

## Author Contributions

EM-M and JS-H conceptualized the overall layout and contents of the review. EM-M, JS-H, NT, RM-G, and SL-S collected and analyzed the literature, compiled the initial draft, and designed the Figures. EM-M and SL-S summarized the Tables. RP-S pre-checked the collected literature and drafted the manuscript. EM-M, JS-H, and RP-S made revisions and final editing of the final version. JS-H processed for publication. All authors read and approved the final manuscript.

## Conflict of Interest

The authors declare that the research was conducted in the absence of any commercial or financial relationships that could be construed as a potential conflict of interest.
